# Comparative Brain and Central Nervous System Tumor Incidence and Survival between the United States and Taiwan Based on Population-Based Registry

**DOI:** 10.3389/fpubh.2016.00151

**Published:** 2016-07-21

**Authors:** Li-Nien Chien, Haley Gittleman, Quinn T. Ostrom, Kuo-Sheng Hung, Andrew E. Sloan, Yi-Chen Hsieh, Carol Kruchko, Lisa R. Rogers, Ye-Fan Glavin Wang, Hung-Yi Chiou, Jill S. Barnholtz-Sloan

**Affiliations:** ^1^College of Management, School of Health Care Administration, Taipei Medical University, Taipei, Taiwan; ^2^Comprehensive Cancer Center of Taipei Medical University, Taipei, Taiwan; ^3^Case Comprehensive Cancer Center, School of Medicine, Case Western Reserve University, Cleveland, OH, USA; ^4^Central Brain Tumor Registry of the United States, Hinsdale, IL, USA; ^5^Department of Neurosurgery, Clinical Research Center, Taipei Medical University-Wan Fang Hospital, Taipei, Taiwan; ^6^Graduate Institute of Injury Prevention and Control, Taipei Medical University, Taipei, Taiwan; ^7^Brain Tumor and Neuro-Oncology Center, University Hospitals Case Medical Center, Cleveland, OH, USA; ^8^Ph.D. Program for Neural Regenerative Medicine, College of Medical Science and Technology, Taipei Medical University, Taipei, Taiwan; ^9^Department of Family Medicine and Community Health, School of Medicine, Case Western Reserve University, Cleveland, OH, USA; ^10^Taipei Medical University, Taipei, Taiwan; ^11^College of Public Health and Nutrition, School of Public Health, Taipei Medical University, Taipei, Taiwan

**Keywords:** malignant brain and central nervous system tumors, incidence, survival, CBTRUS, Taiwan Cancer Registry

## Abstract

**Purpose:**

Reasons for worldwide variability in the burden of primary malignant brain and central nervous system (CNS) tumors remain unclear. This study compares the incidence and survival of malignant brain and CNS tumors by selected histologic types between the United States (US) and Taiwan.

**Methods:**

Data from 2002 to 2010 were selected from two population-based cancer registries for primary malignant brain and CNS tumors: the Central Brain Tumor Registry of the United States and the Taiwan Cancer Registry. Two registries had similar process of collecting patients with malignant brain tumor, and the quality of two registries was comparative. The age-adjusted incidence rate (IR), IR ratio, and survival by histological types, age, and gender were used to study regional differences.

**Results:**

The overall age-adjusted IRs were 5.91 per 100,000 in the US and 2.68 per 100,000 in Taiwan. The most common histologic type for both countries was glioblastoma (GBM) with a 12.9% higher proportion in the US than in Taiwan. GBM had the lowest survival rate of any histology in both countries (US 1-year survival rate = 37.5%; Taiwan 1-year survival rate = 50.3%). The second largest group was astrocytoma, excluding GBM and anaplastic astrocytoma, with the distribution being slightly higher in Taiwan than in the US.

**Conclusion:**

Our findings revealed differences by histological type and grade of primary malignant brain and CNS tumors between two sites.

## Introduction

Differences in brain and central nervous system (CNS) tumor incidence have been reported between countries worldwide. In general, the incidence of brain and CNS tumors of the Western world is higher than that of the Eastern world (1) and higher in developed countries compared with less developed countries (2, 3). Differences are also seen between ethnic and racial groups within the same country. For example, brain tumors are less common among Asian Americans compared with Whites in the United States (US) and United Kingdom (UK) (4, 5).

Differences in survival of brain and CNS tumors also exist. Studies conducted in the US show that differences in survival are mainly due to the variation in histologic type and grade after adjusting for age, sex, and treatment (6–8). Geographical differences in the delivery of care to cancer patients within the UK might be reflected in differences of long-term survival (9). A comparison study found a higher survival rate among the most common pediatric CNS tumors in the UK compared with those in the US (10); however, the reasons for this difference in observed survival are not known.

The etiology attributed to these differences is not entirely clear. Several factors, such as differences in cancer screening practices, availability and quality of medical treatment, and age distribution, could result in regional differences in the burden of brain cancer (3, 8, 9, 11–14). However, very few studies examine the difference in incidence and survival of brain and CNS tumors directly across the countries (15). Although ethnicity, cultural beliefs, lifestyles, socioeconomic conditions, and health-care systems are quite different between the US and Taiwan, the methods and processes of collecting data on newly diagnosed cancer cases are similar. Investigating the differences and similarities of two heterogeneous groups while controlling the variation in ascertaining cancer cases could help further the knowledge of the epidemiology of malignant brain and CNS tumors. Therefore, the aim of this study is to compare the incidence and survival of malignant brain and CNS tumors by selected histologic types between the US and Taiwan using population-based data from both countries.

## Materials and Methods

### Ethics Statement

This study was approved by the Taipei Medical University Joint Institutional Review Board and conducted under the approval from the University Hospitals Case Medical Center Institutional Review Board. In addition, Central Brain Tumor Registry of the United States (CBTRUS) adheres to a confidentiality agreement per the Centers for Disease Control (CDC)/National Program of Cancer Registries (NPCR). Confidentiality of the data from Taiwan was assured by the data regulations of the Health and Welfare Data Science Center (HWDC), Ministry of Health and Welfare, Executive Yuan, Taiwan.

### Data Sources

Two major data sources were used in this study, compiling all primary malignant brain and CNS tumor diagnosed from 2002 to 2010 from the US and Taiwan. The CBTRUS is very comprehensive and is the largest aggregation of population-based incidence data on primary brain and CNS tumors in the US. CBTRUS contains incidence data from 50 central cancer registries [45 NPCR and 5 Surveillance, Epidemiology, and End Results (SEER)] representing ~98% of the US population (4).

The Taiwan Cancer Registry (TCR) is organized and funded by the Ministry of Health and Welfare of the executive branch of the central government since 1979. Following the enactment of the Cancer Control Act in 2003, all hospitals are mandated to submit cancer data to TCR. Additionally, TCR data are subjected to periodic quality control audits and are processed according to the standard guidelines of the International Agency for Research on Cancer, resulting in 2-year time lag between collection and publication of data. It is also overseen by an advisory board run by the National Public Health Association that works to standardize terminology, coding, and procedures for the registry.

Information on individual survival after diagnosis is not available in the CBTRUS or TCR data sets, so survival information was obtained from 18 SEER registries in the US and the National Death Registry in Taiwan for the same years. The 18 SEER registries cover ~26% of the US population, and the TCR covers ~100% of the Taiwanese population.

### Patient Selection

The same criteria were used to select patients with malignant brain and CNS tumors from both datasets. The cases were restricted to the International Classification for Disease, Oncology, third edition (16) (ICD-O-3) behavior codes (malignant only), and then histology codes as noted. We finally selected the most common histology groupings for inclusion in our analysis, which includes 183,740 (92.82% of total) in the CBTRUS data and 5,855 (92.54% of total) in the TCR in the final analysis. The percentage of microscopically confirmed (MC) cases of malignant brain and CNS tumors was ~90% of both sites in the study period (CBTRUS: 89.48% versus TCR: 89.68%) The detailed information relevant to the quality of two registries were shown in the Table S1 in Supplementary Material.

The 14 histological groups were chosen for comparison: astrocytoma [excluding glioblastoma (GBM) and anaplastic astrocytoma (AA)] (ICD-O-3 histology codes: 9381, 9384, 9400, 9410, 9411, 9420, 9421, 9424), AA (ICD-O-3 histology code: 9401), GBM (ICD-O-3 histology codes: 9440–9442), oligodendroglioma (ICD-O-3 histology code: 9450), anaplastic oligodendroglioma (ICD-O-3 histology codes: 9451, 9460), oligoastrocytic tumors (ICD-O-3 histology code: 9382), ependymal tumors (ICD-O-3 histology codes: 9391–9393), glioma malignant (not otherwise specified, NOS) (ICD-O-3 histology code: 9380), neuronal and mixed neuronal glial tumors (ICD-O-3 histology codes: 8680, 8693, 9505, 9522, 9523), embryonal tumors (ICD-O-3 histology codes: 8963, 9364, 9470–9474, 9480, 9490, 9500, 9501, 9502, 9508), nerve sheath tumors (ICD-O-3 histology codes: 9540, 9560, 9561, 9571), malignant meningioma (ICD-O-3 histology codes: 9530, 9538, 9539), lymphoma and hematopoietic neoplasms (ICD-O-3 histology codes: 9590, 9591, 9596, 9650–9655, 9659, 9661–9667, 9670, 9671, 9673, 9675, 9680, 9684, 9687, 9690, 9691, 9695, 9698, 9699, 9701, 9702, 9705, 9714, 9719, 9727–9729, 9731, 9733, 9734, 9740, 9741, 9750, 9754–9758, 9760, 9823, 9826, 9827, 9832, 9837, 9860, 9861, 9866, 9930), and germ cell tumors, cysts, and heterotopias (ICD-O-3 histology codes: 8020, 8440, 9060, 9061, 9064, 9065, 9070–9072, 9080–9085, 9100, 9101) (Detailed coding is provided in Table S2 in Supplementary Material). These histologic groups are based on definitions used by CBTRUS in their annual statistical report (4).

### Statistical Analysis

In this study, we presented age-adjusted incidence rates (IRs), adjusted to the World Health Organization (WHO) 2000–2025 World Standard Population. We also calculated the IR ratios to compare IRs of selected tumors by country and sex. The age groups were categorized into children (aged 0–14 years), adolescents and young adults (AYA; aged 15–39 years), adults (aged 40–64 years), and the elderly (aged 65 years and above). The 1-, 2-, and 5-year survival rates were computed based on the life-table (actuarial) methods in both registries and represented the percentage of individuals who have survived during a specified time period. Because the prognosis for brain and CNS tumors varies dramatically depending not only on the histologic grade and anatomic location but also on age and sex of patient, IRs by histology, sex, age, and primary site and survival rate by histology were additionally presented to enhance displaying the similarity and dissimilarity between two countries. The analyses of the two registries were done separately while following the same protocol. Analyses of CBTRUS and SEER registry data were conducted using SEER*Stat 8.04 statistical software. Statistical analyses of TCR data were conducted using SAS/STAT, Version 9.3, and the STATA 12 (StataCorp LP, College Station, TX, USA) software packages.

## Results

### Overall Incidence Rates

**Figure 1:**
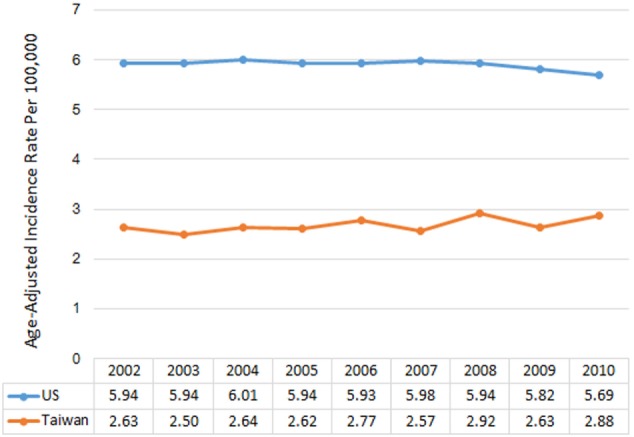
**Annual age-adjusted incidence rates of malignant brain and CNS tumors by year and country, 2002–2010**.

**Figure 2:**
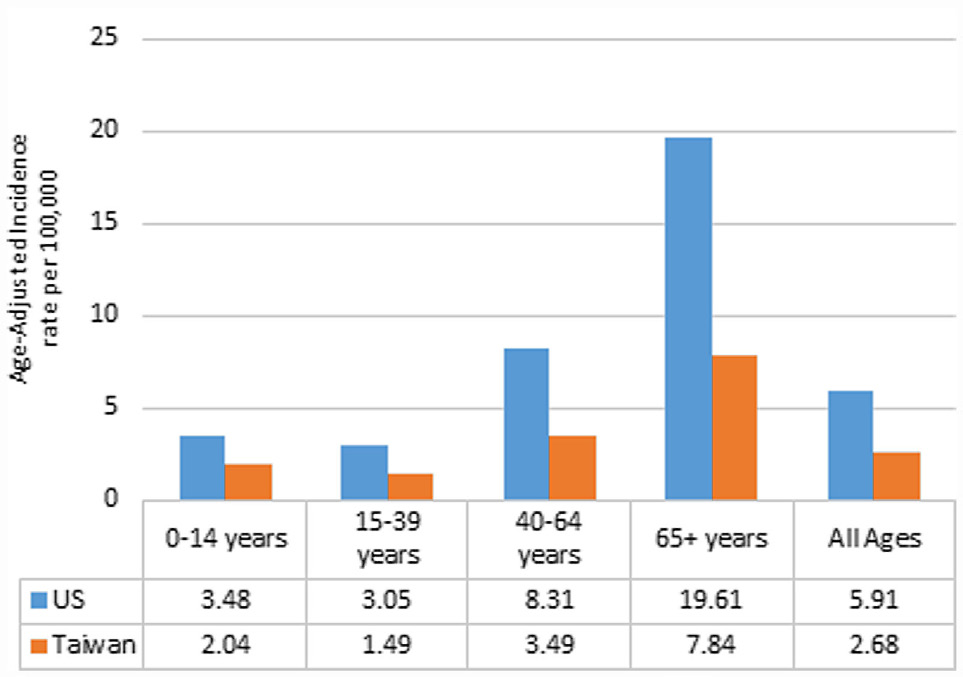
**Annual age-adjusted incidence rates of malignant brain and CNS tumors by age and country, 2002–2010**.

Between 2002 and 2010, there were 183,740 newly diagnosed cases of malignant brain and CNS tumors in the US and 5,855 in Taiwan. IRs by year of diagnosis are presented in Figure 1. The overall age-adjusted IRs were 5.91 per 100,000 in the US and 2.68 per 100,000 in Taiwan. The IRs were relatively constant for each country over the study period, ranging from 5.93 to 6.01 per 100,000 in the US and from 2.63 to 2.92 per 100,000 in Taiwan (Table S3 in Supplementary Material). IRs by age group ranged from 3.05 to 19.61 per 100,000 in the US. The IR for AYA was the lowest followed by children, adults, and the elderly in the US, with a similar pattern in Taiwan (Figure 2).

### Distribution of Tumors by Histology

**Figure 3:**
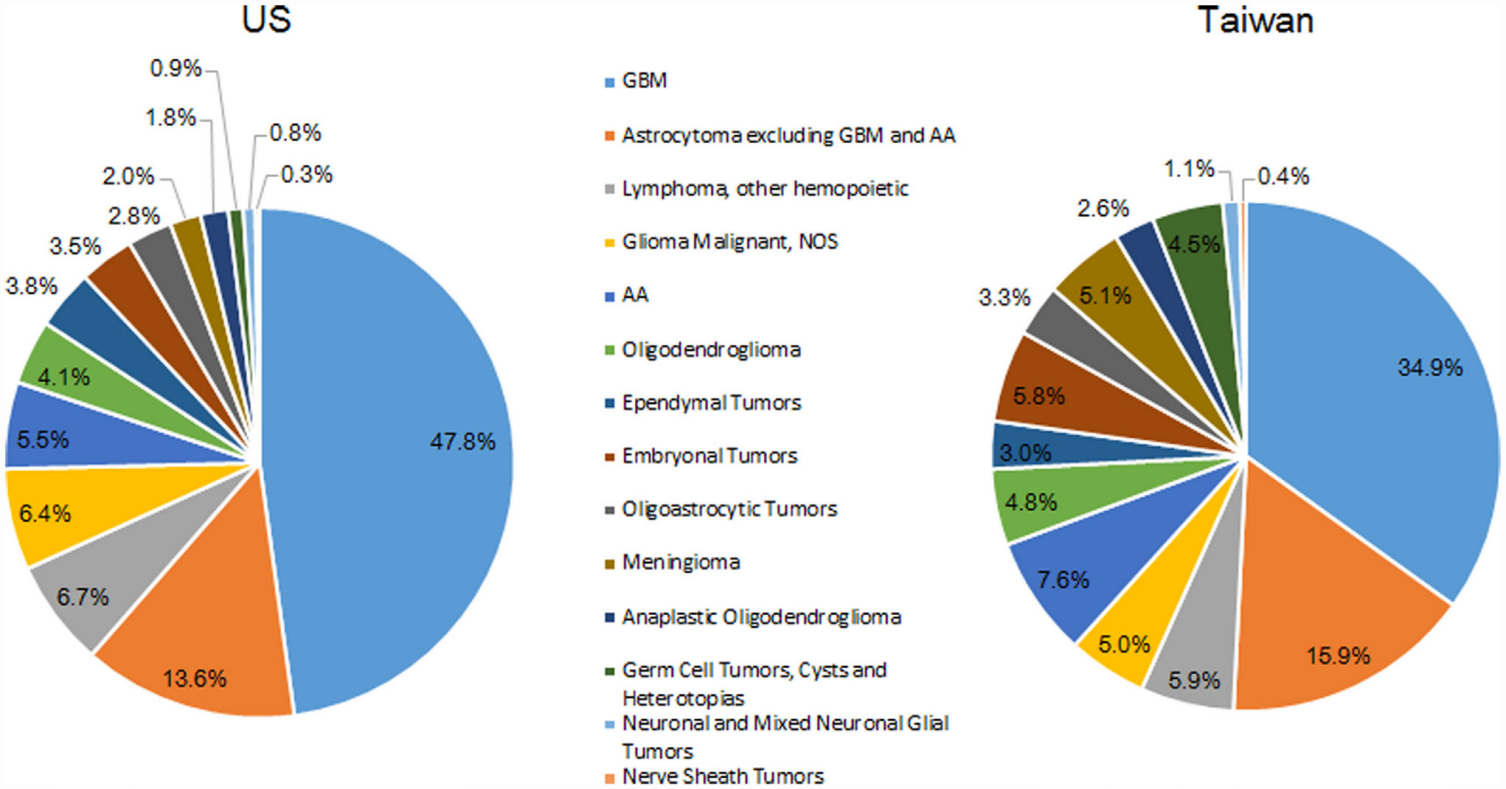
**Distribution of malignant brain and CNS tumors by histology and country, 2002–2010**.

The most common histologic group for both countries was GBM; 47.8% of all tumors in the US and 34.9% of all tumors in Taiwan (Figure 3). The second largest histologic group was astrocytoma (excluding GBM and AA), which made up a slightly higher proportion of all tumors in Taiwan (15.9%) than in the US (13.6%). The third largest histologic group was CNS lymphoma in the US (6.7%) and AA in Taiwan (5.9%).

**Figure 4:**
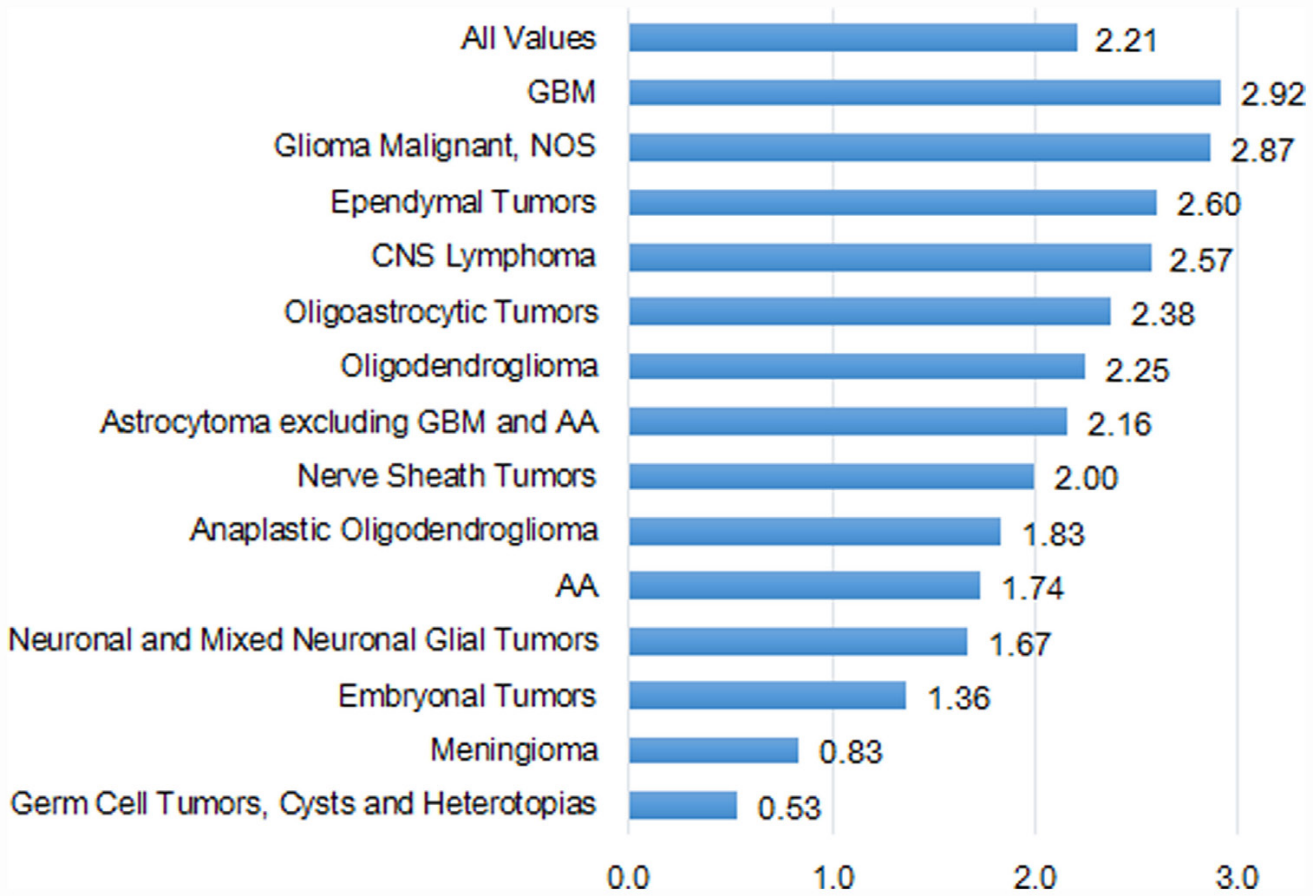
**Incidence rate ratios by country (US:Taiwan) for histologies, 2002–2010**.

### Incidence Rates and Incidence Rate Ratio

Overall, the IRs by histologic groups were significantly greater in the US compared with Taiwan (Table S4 in Supplementary Material). The IR of GBM was 2.9 times in the US (2.48 per 100,000) as compared with Taiwan (0.85 per 100,000). The second highest histologic group was astrocytoma (excluding GBM and AA) in both the US (0.95 per 100,000) and Taiwan (0.44 per 100,000) (Table S4 in Supplementary Material).

The IRs by histologic group also varied based on age group, and these results are shown in Tables S5A,B in Supplementary Material. The most common childhood tumor was the astrocytoma group (excluding GBM and AA) in the US, but in Taiwan it was the embryonal tumor group. For AYA, the most common tumor group was astrocytoma (excluding GBM and AA) in the US as well as in Taiwan. GBM was the most common histologic group in adults and the elderly in both countries.

**Figure 5:**
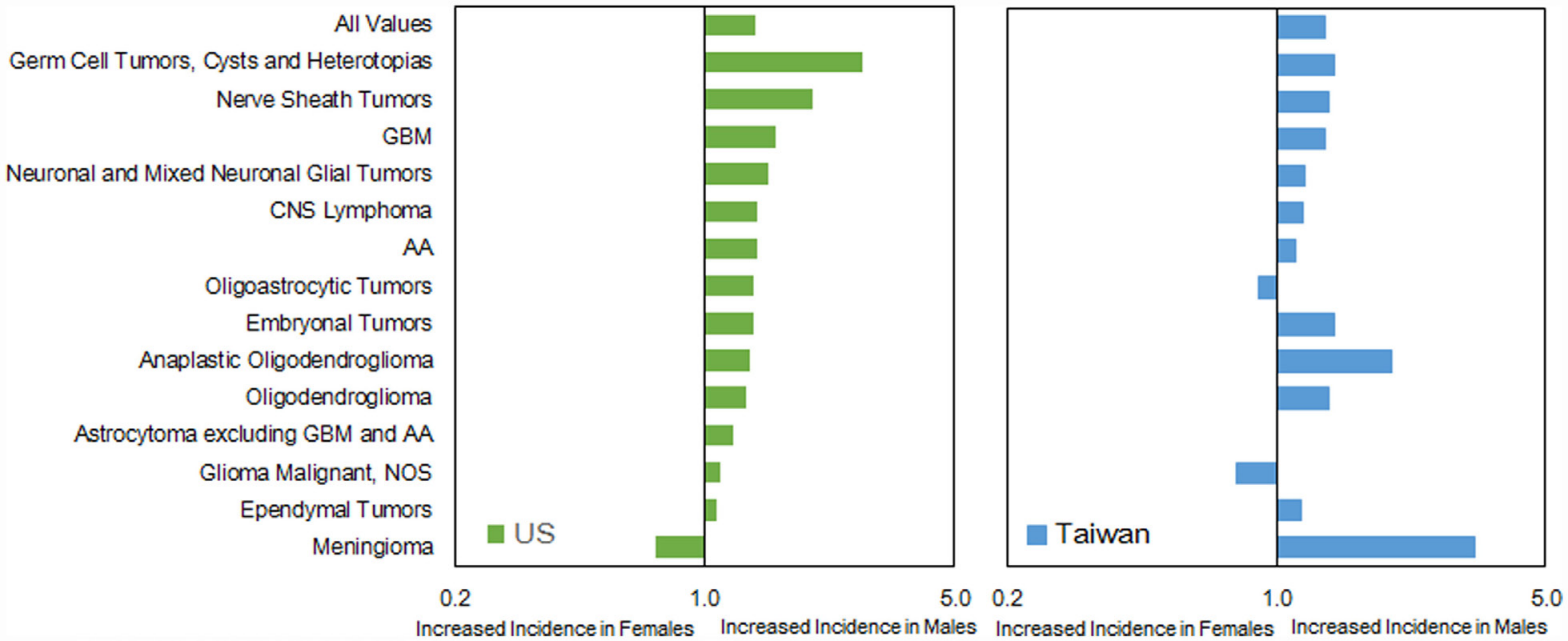
**Incidence rate ratios by sex (male:female) and country for histologies, 2002–2010**.

The IRs by histology were higher in males than females in both countries, except for oligodendroglioma, ependymal tumors, glioma malignant, NOS, nerve sheath tumors, and meningioma in the US (Table S6 in Supplementary Material). The IR ratio (male:female) was similar (1.18 in US versus 1.34 in Taiwan) (Figure 5). In general, the pattern of male:female ratio of selected histological group was quite similar between two registries; however, we observed a greater variation in Taiwan.

In the US, the IRs by primary site were highest for tumors located in the frontal lobe (1.34 per 100,000), followed by tumors located in other brain (i.e., all other sites within the brain), temporal lobe, parietal lobe, and the other parts of brain and CNS (Table S7 in Supplementary Material). In Taiwan, the IRs were highest for tumors located in other brain (0.70 per 100,000), followed by tumors located in the frontal lobe, temporal lobe, and cerebrum.

### Survival Rates by Histology

In general, the overall survival rates for selected tumors were lower in the US than in Taiwan. However, there was a large variation in survival depending upon tumor histology (Table 1). For example, GBM had the lowest 1-year survival rate in both countries (37.5% in the US and 50.3% in Taiwan), and CNS lymphoma was the second lowest in US but the third lowest in Taiwan (1-year survival rate of 51.4% in the US and 60.9% in Taiwan).

**Table 1:** **The 1-, 2-, and 5-year survival^a^ for malignant brain and CNS tumors by histology and country**.

	US^b^				Taiwan			
		1-Year	2-Year	5-Year		1-Year	2-Year	5-Year
				
Histology	*N*	Rate (95% CI)	Rate (95% CI)	Rate (95% CI)	*N*	Rate (95% CI)	Rate (95% CI)	Rate (95% CI)
Neuronal and mixed neuronal glial tumors	82	94.7 (86.6–98.0)	85.7 (74.9–92.0)	71.3 (57.3–81.5)	63	87.3 (76.2–93.4)	71.4 (58.6–80.9)	50.7 (37.4–62.6)
Ependymal tumors	1,781	93.8 (92.6–94.9)	89.3 (87.7–90.8)	82.0 (79.8–84.1)	177	88.1 (82.4–92.1)	83.1 (76.7–87.8)	69.0 (61.3–75.4)
Oligodendroglioma	2,018	93.8 (92.6–94.8)	89.6 (88.1–90.9)	78.7 (76.5–80.8)	283	91.9 (88.0–94.5)	83.0 (78.1–86.9)	70.3 (64.5–75.4)
Germ cell tumors, cysts, and heterotopias	556	91.5 (88.8–93.6)	89.0 (86.0–91.5)	83.8 (79.8–87.0)	263	92.0 (88.0–94.7)	87.1 (82.4–90.6)	81.2 (75.7–85.6)
Oligoastrocytic tumors	1,345	88.1 (86.2–89.8)	77.8 (75.3–80.1)	62.8 (59.5–65.9)	194	89.7 (84.5–93.2)	77.3 (70.8–82.6)	61.9 (54.3–68.6)
Anaplastic oligodendroglioma	797	83.6 (80.8–86.1)	72.2 (68.8–75.4)	54.6 (50.4–58.6)	150	82.7 (75.6–87.9)	62.0 (53.7–69.2)	35.4 (27.3–43.7)
Embryonal tumors	1,738	81.6 (79.7–83.4)	72.5 (70.2–74.7)	60.9 (58.1–63.5)	342	80.7 (76.1–84.5)	69.3 (64.1–73.9)	54.1 (48.6–59.4)
Meningioma	684	79.3 (76.0–82.2)	70.9 (67.2–74.3)	59.4 (55.2–63.4)	299	81.9 (77.1–85.9)	66.9 (61.2–71.9)	49.9 (43.9–55.6)
Nerve sheath tumors	117	78.3 (69.4–84.9)	71.4 (61.8–79.0)	68.2 (58.3–76.2)	23	60.9 (38.3–77.4)	56.5 (34.3–73.8)	52.2 (30.5–70.0)
Astrocytoma excluding GBM and AA	4,101	73.4 (72.0–74.8)	62.9 (61.3–64.4)	48.8 (47.0–50.6)	929	76.0 (73.1–78.6)	63.6 (60.4–66.6)	47.8 (44.5–51.1)
AA	2,313	63.2 (61.1–65.1)	44.8 (42.6–47.0)	27.7 (25.5–29.9)	447	67.8 (63.2–71.9)	45.0 (40.3–49.5)	22.1 (18.1–26.3)
Glioma malignant, NOS	2,821	62.5 (60.6–64.3)	51.9 (49.9–53.8)	44.6 (42.6–46.7)	293	63.8 (58.0–69.0)	43.3 (37.6–48.9)	32.5 (27.1–38.0)
CNS lymphoma	2,898	51.4 (49.5–53.2)	42.6 (40.7–44.5)	31.5 (29.5–33.4)	347	63.1 (57.8–68.0)	47.6 (42.2–52.7)	22.3 (17.8–27.1)
GBM	19,893	37.5 (36.8–38.2)	15.7 (15.2–16.3)	5.0 (4.6–5.4)	2,045	50.3 (48.1–52.4)	24.0 (22.1–25.8)	9.8 (8.5–11.2)
All values	41,144	56.5 (56.0–57.0)	41.1 (40.6–41.6)	30.0 (29.5–30.5)	5,855	68.1 (66.9–69.3)	49.9 (48.6–51.2)	34.3 (33.1–35.6)

*^a^Rates are sorted from the largest to the smallest based on the 1-year US data*.

^b^Estimated by CBTRUS using Surveillance, Epidemiology, and End Results (SEER) Program (www.seer.cancer.gov) SEER*Stat Database: incidence – SEER 18 Regs Research Data + Hurricane Katrina Impacted Louisiana Cases, November 2013 Sub (1973–2011 varying) – Linked To County Attributes – Total US, 1969–2012 Counties, National Cancer Institute, DCCPS, Surveillance Research Program, Cancer Statistics Branch, released April 2014, based on the November 2013 submission.

*AA, anaplastic astrocytoma; CI, confidence interval; CNS, central nervous system; GBM, glioblastoma; NOS, not otherwise specified*.

## Discussion

Our findings revealed that there were some differences in the incidence of and survival after diagnosis with malignant brain and CNS tumors in the US compared with that in Taiwan. We found the age-adjusted incidence of malignant brain and CNS tumors in the US to be greater than that in Taiwan; however, the survival rate in the US was slightly lower than that in Taiwan. From 2002 to 2010, there were no significant changes in IRs in the US and Taiwan. The distribution by histologic type varied between the two countries, although GBM had the highest frequency in both countries. The overall IR ratio by sex was similar in both countries, although the male:female ratios were not consistent in certain histologic groups. Incidence by age and histology was also very similar in both countries, except for the most common tumor in children: astrocytoma (excluding GBM and AA) in the US while embryonal tumors in Taiwan.

Regional differences in IRs have been reported for many other types of cancers (11, 12, 15, 17–19) and brain tumor (14). Most of these studies suggested the difference was a result of a combination effect of differing in screening practices, disparities in lifestyle, race, and hereditary factors. In this study, the lower age-adjusted IRs of malignant brain and CNS tumors in Taiwan was less likely due to differences in imaging diagnostic techniques as the standards for imaging for brain and CNS tumors was the same in both countries. Many epidemiologic studies have attempted to identify the potential risk factors for brain tumors (9, 14, 20). Only ionizing radiation exposure and certain genetic syndromes were well-defined risk factors for malignant glioma, and history of allergy provides some decrease in risk, but there was no established risk factor that accounts for a majority of tumors (21). This current study tended minimizes the bias due to diagnostic practice, cases ascertainment, and access to care of brain tumor cases as compared the US with Taiwan; however, we still found the dissimilarity of cancer incidence between two countries. Further research is required to investigate whether the difference was due to genetic syndromes or other reasons.

The male:female IR ratio revealed some important information. For example, the ratio of meningioma was smaller in US (0.91) than that in Taiwan (0.79). Several factors have been explored as potential risk factors for meningioma, including exogenous sex hormone exposure (22–25). Though this association was unclear and required further investigation, this or other currently unknown environmental or behavioral factors could be involved in this difference in incidence between these two populations. Additionally, the data in Taiwan were restricted to a relatively small sample in each specific histological group that might result in a bigger fluctuation of rate ratio (Table S4 in Supplementary Material).

Ancestral variation might account for differences in incidence and survival between the US and Taiwan. Many population-based studies have addressed racial and ethnic differences in cancer incidence and survival and found that race/ethnicity was a major determinant in the incidence and survival in malignant brain and CNS tumors (7, 26–29). To further explore this difference, we limited our analysis to Asian/Pacific Islanders (APIs) in the US and found that the age-adjusted IR of malignant brain and CNS tumors for APIs was 4.66 per 100,000, which was still greater than that in Taiwan.

Survival after being diagnosed with malignant brain and CNS tumors varied significantly depending on the histologic type and treatment. We found the overall survival rate in Taiwan to be higher than that in the US, which could be partially attributed to the larger proportion of GBM in the US. Median survival for GBM was ~12–14 months, but the survival rate for those with GBM was lower in the US compared with Taiwan. Access to health care could also be a factor in survival outcome for brain tumor patients in the US. Although provision of health care in the US and Taiwan was quite similar, the insurance coverage was 99% in Taiwan because of the national health insurance system (30), whereas the insurance coverage was only 84% in the US (31). Several studies have shown that lower cancer survival rates were associated with not having health insurance (32–34). Recently, the Affordable Care Act has significantly decreased the number of citizens without health insurance in the US (35); however, whether it would also increase cancer survival rates was yet to be determined.

Several limitations were needed to address. First, we selected the most common fourteen histology groupings in the final analysis since the CBTRUS policy was to suppress data presentation for cells with counts of <16 in part because the reliability of such rates would be questionable. In Taiwan, the cell with count of <2 was restricted to report under the HWDC regulation. While comparisons between reports were not always straightforward, these data represented the majority of brain tumors in both the US and Taiwan for the period examined. Second, although the relative survival was a standard method to estimate cancer survival in the absence of other cause of death (36), this study used the observed survival rate in both registries due to the difficulty to obtain the relative cancer survival in two different registries. Additionally, intraobserver variability among histopathologists might cause skewed distributions of histopathological subgroups and their subsequent analyses in both countries, although we did not see the significant different between the diagnostic confirmation between two sites.

## Conclusion

Our findings revealed differences by histological type and grade of primary malignant brain and CNS tumors between two sites. Comparative studies that minimizing the bias due to case ascertainment and inconsistent report were vital in our understanding of brain and CNS tumor burden in these two countries. Further studies are required to examine the factors attributed the dissimilarity.

## Author Contributions

L-NC, HG, and QO participated in the design of the study and performed the statistical analysis. K-SH, AS, Y-CH, CK, LR, Y-FW, H-YC, and JB-S conceived of the study, participated in its design and coordination, and helped to draft the manuscript. All authors read and approved the final manuscript.

## Conflict of Interest Statement

The authors report no conflict of interest concerning the materials or methods used in this study or the findings specified in this paper.
