# Supplementary material for: Comparative Brain and Central Nervous System Tumor Incidence and Survival between the United States and Taiwan Based on Population-Based Registry
Source: Front Public Health. 2016 Jul 21;4:151. doi: 10.3389/fpubh.2016.00151 (PMC4954825; doi:10.3389/fpubh.2016.00151)
Supplement: Supplementary file 1 [file Table_1.DOC]

Supplementary Table 1: The Distribution of Diagnostic Confirmation Status for Malignant Brain and CNS Tumors by Country, 2002-2010

|  | United States (CBTRUS) | | Taiwan (TCR) | |
| --- | --- | --- | --- | --- |
| Diagnostic Confirmation | Frequency | Proportion of total | Frequency | Proportion of total |
| Microscopically confirmed | 164,419 | 89.48% | 5251 | 89.68% |
| Positive laboratory test/marker study | 69 | 0.04% | 6 | 0.10% |
| Direct visualization without microscopic confirmation | 161 | 0.09% | 3 | 0.05% |
| Radiography without microscopic confirmation | 16,218 | 8.83% | 515 | 8.80% |
| Clinical diagnosis only | 752 | 0.41% | 80 | 1.37% |
| Unknown | 2,121 | 1.15% | 0 | 0% |
| Total | 183,740 | 100% | 5855 | 100% |

Abbreviations: CBTRUS, Central Brain Tumor Registry of the United States TCR, Taiwan Cancer Registry

| Supplementary Table 2: The ICD-O-3 Histology Code for the selected Malignant Brain and CNS tumors | | |
| --- | --- | --- |
| Major histological group | ICD-O-3* Histology Code |  |
| Astrocytoma (excluding GBM and AA) | 9381/3, 9384/3, 9400/3, 9410/3, 9411/3, 9420/3, 9421/3, 9424/3 |  |
| AA | 9401/3 |  |
| GBM | 9440/3, 9441/3, 9442/3 |  |
| Oligodendroglioma | 9450/3 |  |
| Anaplastic Oligodendroglioma | 9451/3, 9460/3 |  |
| Oligoastrocytic Tumors | 9382/3 |  |
| Ependymal Tumors | 9391/3, 9392/3, 9393/3 |  |
| Glioma Malignant, NOS | 9380/3 |  |
| Neuronal and Mixed Neuronal-Glial Tumors | 8680/3, 8693/3, 9505/3, 9522/3, 9523/3 |  |
| Embryonal Tumors | 8963/3, 9364/3, 9470/3, 9471/3, 9472/3, 9473/3, 9474/3, 9480/3, 9490/3, 9500/3, 9501/3, 9502/3, 9508/3 |  |
| Nerve Sheath Tumors | 6163/3, 6243/3, 6247/3, 6287/3 |  |
| Meningioma | 9530/3, 9538/3, 9539/3 |  |
| CNS Lymphoma | 9590/3, 9591/3, 9596/3, 9650/3, 9651/3, 9652/3, 9653/3, 9654/3, 9655/3, 9659/3, 9661/3, 9662/3, 9663/3, 9664/3, 9665/3, 9667/3, 9670/3, 9671/3, 9673/3, 9675/3, 9680/3, 9684/3, 9687/3, 9690/3, 9691/3, 9695/3, 9698/3, 9699/3, 9701/3, 9702/3, 9705/3, 9714/3, 9719/3, 9728/3, 9729/3, 9727/3, 9731/3, 9733/3, 9734/3, 9740/3, 9741/3, 9750/3, 9754/3, 9755/3, 9756/3, 9757/3, 9758/3, 9760/3, 9823/3, 9826/3, 9827/3, 9832/3, 9837/3, 9860/3, 9861/3, 9866/3, 9930/3 |  |
| Germ Cell Tumors, Cysts and Heterotopias | 8020/3, 8440/3, 9060/3, 9061/3, 9064/3, 9065/3, 9070/3, 9071/3, 9072/3, 9080/3, 9081/3, 9082/3, 9083/3, 9084/3, 9085/3, 9100/3, 9101/3 |  |
| * International Classification of Diseases for Oncology, 3rd Edition, 2000. World Health Organization, Geneva, Switzerland. | |  |

Abbreviation: AA, Anaplastic Astrocytoma; GBM, Glioblastoma; NOS, not otherwise specified

| Supplementary Table3: Average Age-Adjusted Incidence Rate by Country, 2002-2010 | | | | | | | |
| --- | --- | --- | --- | --- | --- | --- | --- |
|  | US(CBTRUS) | | |  | Taiwan (TCR) | | |
| Year | N | Rate | 95% CI |  | N | Rate | 95% CI |
| 2002 | 19,288 | 5.94 | (5.86-6.03) |  | 604 | 2.63 | (2.42-2.83) |
| 2003 | 19,600 | 5.94 | (5.85-6.03) |  | 586 | 2.50 | (2.30-2.70) |
| 2004 | 20,097 | 6.01 | (5.92-6.09) |  | 628 | 2.64 | (2.43-2.85) |
| 2005 | 20,287 | 5.94 | (5.86-6.03) |  | 625 | 2.62 | (2.41-2.83) |
| 2006 | 20,454 | 5.93 | (5.85-6.01) |  | 661 | 2.77 | (2.55-2.98) |
| 2007 | 20,900 | 5.98 | (5.89-6.06) |  | 649 | 2.57 | (2.37-2.77) |
| 2008 | 21,132 | 5.94 | (5.86-6.02) |  | 723 | 2.92 | (2.70-3.14) |
| 2009 | 21,112 | 5.82 | (5.73-5.90) |  | 653 | 2.63 | (2.42-2.84) |
| 2010 | 20,870 | 5.69 | (5.61-5.77) |  | 726 | 2.88 | (2.66-3.10) |
| Total | 183,740 | 5.91 | (5.88-5.93) |  | 5,855 | 2.68 | (2.61-2.75) |
| Rates are per 100,000 and age-adjusted to the World (WHO 2000-2025) Standard Million (single ages to 84) standard rates.  Abbreviation: CI, Confidence Interval; CBTRUS, Central Brain Tumor Registry of the United States TCR, Taiwan Cancer Registry | | | | | | | |

| Supplementary Table4: Average Age-Adjusted Incidence Rate^#^ By Histology | | | | | | | | | |
| --- | --- | --- | --- | --- | --- | --- | --- | --- | --- |
|  | US | | | |  | Taiwan | | | |
| Major histological group | N | % | Rate | 95% CI |  | N | % | Rate | 95% CI |
| GBM | 87,867 | 0.48 | 2.48 | (2.46-2.49) |  | 2,045 | 0.35 | 0.85 | (0.82-0.89) |
| Astrocytoma excluding GBM and AA | 25,020 | 0.14 | 0.95 | (0.94-0.97) |  | 929 | 0.16 | 0.44 | (0.41-0.47) |
| Glioma Malignant, NOS | 11,800 | 0.06 | 0.43 | (0.42-0.44) |  | 293 | 0.05 | 0.15 | (0.13-0.17) |
| CNS Lymphoma | 12,402 | 0.07 | 0.36 | (0.35-0.36) |  | 347 | 0.06 | 0.14 | (0.13-0.16) |
| AA | 10,133 | 0.06 | 0.33 | (0.32-0.34) |  | 447 | 0.08 | 0.19 | (0.18-0.21) |
| Embryonal Tumors | 6,423 | 0.03 | 0.30 | (0.29-0.30) |  | 342 | 0.06 | 0.22 | (0.20-0.25) |
| Oligodendroglioma | 7,509 | 0.04 | 0.27 | (0.26-0.27) |  | 283 | 0.05 | 0.12 | (0.11-0.14) |
| Ependymal Tumors | 6,957 | 0.04 | 0.26 | (0.25-0.27) |  | 177 | 0.03 | 0.10 | (0.08-0.11) |
| Oligoastrocytic Tumors | 5,193 | 0.03 | 0.19 | (0.18-0.19) |  | 194 | 0.03 | 0.08 | (0.07-0.10) |
| Anaplastic Oligodendroglioma | 3,232 | 0.02 | 0.11 | (0.10-0.11) |  | 150 | 0.03 | 0.06 | (0.05-0.07) |
| Meningioma | 3,602 | 0.02 | 0.10 | (0.10-0.10) |  | 299 | 0.05 | 0.12 | (0.11-0.14) |
| Germ Cell Tumors, Cysts and Heterotopias | 1,724 | 0.01 | 0.08 | (0.07-0.08) |  | 263 | 0.04 | 0.15 | (0.13-0.17) |
| Neuronal and Mixed Neuronal Glial Tumors | 1,398 | 0.01 | 0.05 | (0.05-0.05) |  | 63 | 0.01 | 0.03 | (0.02-0.03) |
| Nerve Sheath Tumors | 480 | 0.00 | 0.02 | (0.01-0.02) |  | 23 | 0.00 | 0.01 | (0.01-0.02) |
| All Values | 183,740 | 1.00 | 5.91 | (5.88-5.93) |  | 5,855 | 1.00 | 2.68 | (2.61-2.75) |
|  |  |  |  |  |  |  |  |  |  |
| Rates are per 100,000 and age-adjusted to the World (WHO 2000-2025) Std Million (single ages to 84) standard rates. | | | | | | | | | |
| ^#^Rates are sorted from the largest to the smallest based on the US data  Abbreviation: AA, Anaplastic Astrocytoma; CI: confidence interval; GBM: Glioblastoma | | | |  |  |  |  |  |  |

| Supplementary Table 5(a): Average Age-Adjusted Incidence Rate By Histology and Age (US, CBTRUS), 2002-2010 | | | | | | | | | | | | | | | |
| --- | --- | --- | --- | --- | --- | --- | --- | --- | --- | --- | --- | --- | --- | --- | --- |
|  | 0-14 | | |  | 15-39 | | |  | 40-64 | | |  | 65+ | | |
| Major histological group | N | Rate | 95% CI |  | N | Rate | 95% CI |  | N | Rate | 95% CI |  | N | Rate | 95% CI |
| Astrocytoma excluding GBM and AA | 6,571 | 1.23 | (1.20-1.26) |  | 7,368 | 0.81 | (0.79-0.83) |  | 6,976 | 0.81 | (0.79-0.83) |  | 4,105 | 1.25 | (1.21-1.29) |
| AA | 420 | 0.08 | (0.07-0.09) |  | 2,345 | 0.25 | (0.24-0.26) |  | 4,448 | 0.52 | (0.24-0.26) |  | 2,920 | 0.91 | (0.87-0.94) |
| GBM | 706 | 0.13 | (0.12-0.14) |  | 4,107 | 0.44 | (0.42-0.45) |  | 39,778 | 4.54 | (0.42-0.45) |  | 43,276 | 13.12 | (13.00-13.25) |
| Oligodendroglioma | 259 | 0.05 | (0.04-0.05) |  | 2,906 | 0.31 | (0.30-0.32) |  | 3,629 | 0.43 | (0.30-0.32) |  | 715 | 0.23 | (0.21-0.25) |
| Anaplastic Oligodendroglioma | 45 | 0.01 | (0.01-0.01) |  | 878 | 0.09 | (0.09-0.10) |  | 1,783 | 0.21 | (0.09-0.10) |  | 526 | 0.17 | (0.16-0.19) |
| Oligoastrocytic Tumors | 156 | 0.03 | (0.02-0.03) |  | 2,154 | 0.23 | (0.22-0.24) |  | 2,300 | 0.27 | (0.22-0.24) |  | 583 | 0.19 | (0.17-0.20) |
| Ependymal Tumors | 1,414 | 0.27 | (0.25-0.28) |  | 1,877 | 0.20 | (0.19-0.21) |  | 2,817 | 0.33 | (0.19-0.21) |  | 849 | 0.28 | (0.26-0.30) |
| Glioma Malignant, NOS | 3,762 | 0.71 | (0.69-0.73) |  | 2,110 | 0.23 | (0.22-0.24) |  | 2,612 | 0.30 | (0.22-0.24) |  | 3,316 | 0.90 | (0.87-0.93) |
| Neuronal and Mixed Neuronal Glial Tumors | 134 | 0.02 | (0.02-0.03) |  | 324 | 0.04 | (0.03-0.04) |  | 644 | 0.07 | (0.03-0.04) |  | 296 | 0.09 | (0.08-0.10) |
| Embryonal Tumors | 4,205 | 0.79 | (0.77-0.82) |  | 1,571 | 0.17 | (0.17-0.18) |  | 515 | 0.06 | (0.17-0.18) |  | 132 | 0.04 | (0.03-0.05) |
| Nerve Sheath Tumors | 22 | 0.00 | (0.00-0.01) |  | 125 | 0.01 | (0.01-0.02) |  | 224 | 0.03 | (0.01-0.02) |  | 109 | 0.03 | (0.03-0.04) |
| Meningioma | 40 | 0.01 | (0.01-0.01) |  | 285 | 0.03 | (0.03-0.03) |  | 1,443 | 0.17 | (0.03-0.03) |  | 1,834 | 0.52 | (0.50-0.54) |
| CNS Lymphoma | 90 | 0.02 | (0.01-0.02) |  | 1,166 | 0.12 | (0.12-0.13) |  | 5,007 | 0.58 | (0.12-0.13) |  | 6,139 | 1.87 | (1.82-1.92) |
| Germ Cell Tumors, Cysts and Heterotopias | 757 | 0.14 | (0.13-0.15) |  | 907 | 0.10 | (0.10-0.11) |  | 50 | 0.01 | (0.10-0.11) |  | - | - | - |
| All Values | 18,581 | 3.48 | (3.43-3.53) |  | 28,123 | 3.05 | (3.01-3.09) |  | 72,226 | 8.31 | (3.01-3.09) |  | 64,810 | 19.61 | (19.45-19.76) |
| Rates are per 100,000 and age-adjusted to the World (WHO 2000-2025) Standard Million (single ages to 84) standard rates. | | | | | | | | | | | | | | | |
| - Indicated the number of sample less than 16 | | | | | | | | | | | | | | | |
| Abbreviation: AA, Anaplastic Astrocytoma; CBTRUS, Central Brian Tumor Registry of the United States; CI, Confidence Interval; GBM, Glioblastoma; NOS, not otherwise specified | | | | | | | | | | | | | | | |

| Supplementary Table 5(b): Average Age-Adjusted Incidence Rate By Histology and Age (Taiwan, TCR), 2002-2010 | | | | | | | | | | | | | | | |
| --- | --- | --- | --- | --- | --- | --- | --- | --- | --- | --- | --- | --- | --- | --- | --- |
|  | 0-14 | | |  | 15-39 | | |  | 40-64 | | |  | 65+ | | |
| Major histological group | N | Rate | 95% CI |  | N | Rate | 95% CI |  | N | Rate | 95% CI |  | N | Rate | 95% CI |
| Astrocytoma (excluding GBM and AA) | 129 | 0.36 | (0.29-0.42) |  | 304 | 0.37 | (0.33-0.41) |  | 330 | 0.51 | (0.45-0.56) |  | 166 | 0.81 | (0.69-0.93) |
| AA | 32 | 0.08 | (0.05-0.11) |  | 112 | 0.13 | (0.11-0.15) |  | 181 | 0.27 | (0.23-0.31) |  | 122 | 0.60 | (0.49-0.71) |
| GBM | 45 | 0.12 | (0.08-0.15) |  | 222 | 0.26 | (0.23-0.29) |  | 916 | 1.44 | (1.35-1.53) |  | 862 | 4.17 | (3.90-4.45) |
| Oligodendroglioma | - | - | - |  | 112 | 0.13 | (0.11-0.16) |  | 137 | 0.21 | (0.17-0.24) |  | 22 | 0.11 | (0.06-0.15) |
| Anaplastic Oligodendroglioma | - | - | - |  | 34 | 0.04 | (0.03-0.05) |  | 86 | 0.13 | (0.10-0.16) |  | 26 | 0.13 | (0.08-0.18) |
| Oligoastrocytic Tumors | - | - | - |  | 67 | 0.08 | (0.06-0.10) |  | 97 | 0.15 | (0.12-0.18) |  | 20 | 0.10 | (0.05-0.14) |
| Ependymal Tumors | 59 | 0.18 | (0.13-0.22) |  | 47 | 0.06 | (0.04-0.07) |  | 57 | 0.09 | (0.06-0.11) |  | - | - | - |
| Glioma Malignant, NOS | 86 | 0.23 | (0.18-0.28) |  | 63 | 0.08 | (0.06-0.10) |  | 73 | 0.11 | (0.09-0.14) |  | 71 | 0.34 | (0.26-0.42) |
| Neuronal and Mixed Neuronal Glial Tumors | - | - | - |  | 19 | 0.02 | (0.01-0.03) |  | 33 | 0.05 | (0.03-0.07) |  | - | - | - |
| Embryonal Tumors | 228 | 0.65 | (0.57-0.74) |  | 72 | 0.09 | (0.07-0.12) |  | 29 | 0.05 | (0.03-0.06) |  | - | - | - |
| Nerve Sheath Tumors | - | - | - |  | - | - | - |  | - | - | - |  | - | - | - |
| Meningioma | - | - | - |  | - | - | - |  | 155 | 0.24 | (0.20-0.28) |  | 111 | 0.54 | (0.44-0.64) |
| CNS Lymphoma | - | - | - |  | - | - | - |  | 143 | 0.23 | (0.19-0.27) |  | 181 | 0.87 | (0.74-0.99) |
| Germ Cell Tumors, Cysts and Heterotopias | 137 | 0.34 | (0.28-0.39) |  | 118 | 0.16 | (0.13-0.19) |  | - | - | - |  | - | - | - |
| All Values | 751 | 2.04 | (1.90-2.19) |  | 1233 | 1.49 | (1.41-1.57) |  | 2253 | 3.49 | (3.35-3.63) |  | 1618 | 7.84 | (7.47-8.21) |
| Rates are per 100,000 and age-adjusted to the World (WHO 2000-2025) Standard Million (single ages to 84) standard rates. | | | | | | | | | | | | | | | |
| - Indicated the number of sample less than 16 | | | | | | | | | | | | | | | |
| Abbreviation: AA, Anaplastic Astrocytoma; CI, Confidence Interval; GBM, Glioblastoma; NOS, not otherwise specified; TCR, Taiwan Cancer Registry | | | | | | | | | | | | | | | |

| Supplementary Table 6: Average Age-Adjusted Incidence Rate^#^ By Histology and Sex, 2002-2010 | | | | | | | | | | | | | | | | | |
| --- | --- | --- | --- | --- | --- | --- | --- | --- | --- | --- | --- | --- | --- | --- | --- | --- | --- |
|  | US (CBTRUS) | | | | | | | |  | Taiwan (TCR) | | | | | | | |
|  | Male | | |  | Female | | |  |  | Male | | |  | Female | | |  |
| Major histological group | N | Rate | 95% CI |  | N | Rate | 95% CI |  |  | N | Rate | 95% CI |  | N | Rate | 95% CI |  |
| GBM | 87,867 | 2.48 | (2.46-2.49) |  | 37,690 | 1.95 | (1.93-1.97) | * |  | 1,176 | 0.98 | (0.93-1.04) |  | 869 | 0.73 | (0.68-0.78) | * |
| Astrocytoma (excluding GBM and AA) | 25,020 | 0.95 | (0.94-0.97) |  | 11,478 | 0.87 | (0.86-0.89) | * |  | 558 | 0.51 | (0.47-0.56) |  | 371 | 0.36 | (0.32-0.40) | * |
| Glioma Malignant, NOS | 11,800 | 0.43 | (0.42-0.44) |  | 5,832 | 0.41 | (0.40-0.43) |  |  | 172 | 0.17 | (0.14-0.20) |  | 121 | 0.12 | (0.10-0.15) |  |
| CNS Lymphoma | 12,402 | 0.36 | (0.35-0.36) |  | 5,796 | 0.30 | (0.30-0.31) | * |  | 182 | 0.15 | (0.13-0.17) |  | 165 | 0.13 | (0.11-0.16) |  |
| AA | 10,133 | 0.33 | (0.32-0.34) |  | 4,467 | 0.28 | (0.27-0.29) | * |  | 262 | 0.22 | (0.20-0.25) |  | 185 | 0.16 | (0.14-0.19) | * |
| Embryonal Tumors | 6,423 | 0.30 | (0.29-0.30) |  | 2,637 | 0.25 | (0.24-0.26) | * |  | 202 | 0.26 | (0.22-0.29) |  | 140 | 0.19 | (0.16-0.22) |  |
| Oligodendroglioma | 7,509 | 0.27 | (0.26-0.27) |  | 3,352 | 0.23 | (0.23-0.24) |  |  | 155 | 0.13 | (0.11-0.16) |  | 128 | 0.11 | (0.09-0.13) |  |
| Ependymal Tumors | 6,957 | 0.26 | (0.25-0.27) |  | 3,395 | 0.25 | (0.24-0.26) |  |  | 84 | 0.09 | (0.07-0.11) |  | 93 | 0.10 | (0.08-0.12) |  |
| Oligoastrocytic Tumors | 5,193 | 0.19 | (0.18-0.19) |  | 2,224 | 0.16 | (0.15-0.16) | * |  | 106 | 0.09 | (0.07-0.11) |  | 88 | 0.08 | (0.06-0.09) |  |
| Anaplastic Oligodendroglioma | 3,232 | 0.11 | (0.10-0.11) |  | 1,430 | 0.09 | (0.09-0.10) | * |  | 81 | 0.07 | (0.05-0.08) |  | 69 | 0.06 | (0.05-0.07) |  |
| Meningioma | 3,602 | 0.10 | (0.10-0.10) |  | 2,249 | 0.11 | (0.11-0.12) | * |  | 133 | 0.11 | (0.09-0.13) |  | 166 | 0.14 | (0.12-0.16) |  |
| Germ Cell Tumors, Cysts and Heterotopias | 1,724 | 0.08 | (0.07-0.08) |  | 420 | 0.04 | (0.04-0.04) | * |  | 209 | 0.23 | (0.20-0.26) |  | 54 | 0.07 | (0.05-0.09) | * |
| Neuronal and Mixed Neuronal Glial Tumors | 1,398 | 0.05 | (0.05-0.05) |  | 587 | 0.04 | (0.04-0.04) | * |  | 43 | 0.04 | (0.03-0.05) |  | 20 | 0.02 | (0.01-0.02) | * |
| Nerve Sheath Tumors | 480 | 0.02 | (0.01-0.02) |  | 236 | 0.01 | (0.01-0.02) |  |  | - | - | - |  | - | - | - |  |
| All Values | 183,740 | 5.91 | (5.88-5.93) |  | 81,793 | 5.01 | (4.98-5.05) | * |  | 3,374 | 3.07 | (2.97-3.18) |  | 2,481 | 2.29 | (2.19-2.38) | * |
| Rates are per 100,000 and age-adjusted to the World (WHO 2000-2025) Std Million (single ages to 84) standard rates. | | | | | | | | | | |  |  |  |  |  |  |  |
| ^#^Rates are sorted from the largest to the smallest based on the US data | | | | | | | | | | |  |  |  |  |  |  |  |
| * Incidence Rate is significantly different in males and females (based on 95% CI) | | | | | |  |  |  |  |  |  |  |  |  |  |  |  |
| - Indicated the number of sample less than 16 | | | | | |  |  |  |  |  |  |  |  |  |  |  |  |

Abbreviation: AA, Anaplastic Astrocytoma; CBTRUS; Central Brain Tumor Registry of the United States; CI, Confidence Interval; GBM, Glioblastoma; NOS, not otherwise specified; TCR, Taiwan Cancer Registry

| Supplementary Table 7: Average Age-Adjusted Incidence Rate^#^ by Site and Country, 2002-2010 | | | | | | | |
| --- | --- | --- | --- | --- | --- | --- | --- |
|  | US (CBTRUS) | | |  | Taiwan (TCR) | | |
| Site | N | Rate | 95% CI |  | N | Rate | 95% CI |
| Frontal Lobe | 43,091 | 1.34 | (1.33-1.36) |  | 1,191 | 0.51 | (0.48-0.53) |
| Other Brain | 38,026 | 1.17 | (1.15-1.18) |  | 1,593 | 0.7 | (0.67-0.74) |
| Temporal Lobe | 32,254 | 0.97 | (0.96-0.98) |  | 771 | 0.34 | (0.31-0.36) |
| Parietal Lobe | 21,763 | 0.64 | (0.63-0.65) |  | 311 | 0.13 | (0.12-0.15) |
| Cerebellum | 9,456 | 0.41 | (0.40-0.42) |  | 406 | 0.24 | (0.22-0.26) |
| Cerebrum | 8,754 | 0.29 | (0.29-0.30) |  | 535 | 0.25 | (0.23-0.27) |
| Brain Stem | 6,945 | 0.29 | (0.28-0.30) |  | 278 | 0.16 | (0.14-0.18) |
| Spinal Cord and Cauda Equina | 5,684 | 0.2 | (0.19-0.21) |  | 20 | 0.01 | (0.01-0.02) |
| Occipital Lobe | 5,613 | 0.16 | (0.16-0.17) |  | 154 | 0.07 | (0.06-0.08) |
| Meninges | 3,746 | 0.11 | (0.10-0.11) |  | 196 | 0.08 | (0.07-0.09) |
| Ventricle | 2,545 | 0.1 | (0.09-0.10) |  | 130 | 0.07 | (0.05-0.08) |
| Cranial Nerves | 2,053 | 0.09 | (0.09-0.10) |  | 91 | 0.04 | (0.03-0.05) |
| Other Nervous System | 1,668 | 0.05 | (0.05-0.05) |  | 87 | 0.04 | (0.03-0.05) |
| Pineal | 938 | 0.04 | (0.04-0.04) |  | 58 | 0.03 | (0.02-0.04) |
| Olfactory Tumors of the Nasal Cavity | 1,004 | 0.03 | (0.03-0.03) |  | 17 | 0.01 | (0.00-0.01) |
| Pituitary and Craniopharyngeal duct | 200 | 0.01 | (0.01-0.01) |  | 17 | 0.01 | (0.00-0.01) |
| All Values | 183,740 | 5.91 | (5.88-5.93) |  | 5,855 | 2.68 | (2.61-2.75) |
| Rates are per 100,000 and age-adjusted to the World (WHO 2000-2025) Standard Million (single ages to 84) standard rate.  ^#^ Rates are sorted from the largest to the smallest based on the US data  Abbreviation: AA, Anaplastic Astrocytoma; CBTRUS; Central Brain Tumor Registry of the United States; CI, Confidence Interval; GBM, Glioblastoma TCR, Taiwan Cancer Registry | | | | | | | |
